# Violence in Quilombola women living in rural communities in Brazil

**DOI:** 10.11606/s1518-8787.2022056004651

**Published:** 2022-11-18

**Authors:** Thaís Verly Luciano, Beniamino Cislaghi, Raquel Barbosa Miranda, Jerusa Araújo Dias, Ximena Pamela Diaz-Bermudez, Angelica Espinosa Miranda

**Affiliations:** I Universidade Federal do Espírito Santo Centro de Ciências da Saúde Programa de Pós-Graduação em Saúde Coletiva Vitória ES Brasil Universidade Federal do Espírito Santo. Centro de Ciências da Saúde. Programa de Pós-Graduação em Saúde Coletiva. Vitória, ES, Brasil; II London School of Hygiene and Tropical Medicine London United Kingdom London School of Hygiene and Tropical Medicine. London, United Kingdom; III Universidade de Brasília Faculdade de Ciências da Saúde Programa de Pós-Graduação em Saúde Coletiva Brasília DF Brasil Universidade de Brasília. Faculdade de Ciências da Saúde. Programa de Pós-Graduação em Saúde Coletiva. Brasília, DF, Brasil

**Keywords:** Battered Women, Quilombola Communities, Violence Against Women, Domestic Violence, Intimate Partner Violence

## Abstract

**OBJETIVE:**

To estimate the prevalence of psychological, physical, and sexual violence perpetrated against women by their intimate partner (IP) in Quilombola communities located in Espírito Santo State, Brazil.

**METHODS:**

The data is from a population-based cross-sectional study of Quilombola women conducted from 2017 to 2018. In-person interviews collected information on women’s sociodemographic characteristics, behaviors, and their experience of violence perpetrated by their IP. The analysis used chi-square test and hierarchical logistic regression.

**RESULTS:**

219 women (94.8% of the invited ones) agreed to participate in the study. 59.0% (95%CI: 5.25–65.5) reported psychological violence; 41% (95%CI: 34.5–47.5) physical violence; and 8.2% (95%CI: 4.6–11.8) sexual violence. Psychological violence was associated with having three or more sexual partners in life, when compared to those who had up to two partners (p = 0,009), and previous violence involving other people outside of family increased the chance of suffering psychological violence by an IP more than nine times (p ≤ 0.001). Regarding physical violence, the association with use of barrier contraception (p = 0.031) and having a partner with other sexual partners (p = 0.024) were protective factors for IP violence. Having 3 or more sexual partners in the last 12 months (p = 0.006), partner using illicit drugs (p = 0,006), and alcoholism in the family (p = 0,001), increased the chance of suffer physical violence by the partner. Sexual violence perpetrated by the IP was associated with miscarriage (p = 0.016), partner using drugs (p = 0.020), and gynecological symptoms (p = 0.045).

**CONCLUSIONS:**

These results showed the high frequency of intimate partner violence in Quilombola women and highlight the importance of reducing social and race inequities for interrupting the culture of violence against women.

## INTRODUCTION

Intimate partner violence (IPV) causes physical, sexual, or psychological harm. It includes acts of physical aggression, sexual coercion, psychological abuse, and controlling behaviors^1^. Global prevalence of IPV against women is estimated to be 30%, with greater proportions in the regions of Asia and Africa^1^. A multicenter study conducted by World Health Organization (WHO) showed that 13 to 61% of women younger than 50 years reported physical or sexual violence practiced by an intimate partner (IP) at some point in life^2^. Some authors report that this violence occurs unevenly between races, since Black women are more frequently affected by various forms of violence perpetrated by their partners in a context of gender and race opression^3,4^.

A study conducted with women of African descent showed that 30% reported cumulative violence (psychological, physical, and sexual) by an IP^5^. More than half of these women had clinically significant depressive symptoms and 35% had post-traumatic stress disorder^5^. Nadda and collaborators reported 37% of IPV in India and demonstrated that women living in rural areas were significantly more likely to experience a situation of violence than women living in urban areas^6^. Regarding sexual violence, a multicenter study conducted in Asia and the Pacific, in 2017, identified a high frequency of IPV, pointing out risk factors such as poverty, economic dependence on the partner, risky behavior of the partner, use of alcohol and drugs, and experiencing some type of abuse in childhood^7^. Brazilian studies also report significant rates of sexual violence by an IP^4,8,9^. This cycle of violence is difficult to break, since the contact with the aggressor is daily and, in most cases, there is economic and emotional dependence.

The Quilombola population includes descendants of African people who were enslaved and brought to Brazil and who, over time, escaped slavery^10^. Their name is derived from the word *quilombo*, describing the formation of family groups that resisted the slave system in Brazil, and their ethnic and cultural identity distinguish them from other Black communities in the country^1^. Quilombolas organize themselves in isolated rural communities. They are considered a vulnerable population, both socially and economically, also due to restricted access to education, basic sanitation, and adequate health services^11^.

The historical and social vulnerability of the Black population have been affected by violence in Brazil and the specificities of Quilombola women are important public health issues. Quilombola women have increasingly occupied spaces of leadership within their community. On the other hand, women’s increased leadership exposed them to higher risk of domestic violence by threatening traditional gender roles^12^. In many cases, the idea of protecting the community tradition enables socially constructed customs that perpetuates psychological, moral, patrimonial, physical, and sexual violence, often triggered by their own partners^12^.

This study aims to identify the prevalence of psychological, physical, and sexual violence caused by one’s intimate partner and its associated factors in Quilombola women living in rural communities from two municipalities in Brazil.

## METHODS

A cross-sectional study conducted with Quilombola women living in rural communities in the municipalities of São Mateus and Conceição da Barra, in the State of Espirito Santo, Brazil. Women who had at least one intimate partner throughout their lives, and who lived in Quilombola communities were invited to participate in the study. A total 25 communities were encompassed, 17 in São Mateus and 8 in Conceição da Barra. This study is part of the project about sexual and reproductive health of Quilombola women in Espirito Santo, Brazil.

Data were collected from June 2017 to August 2018, using a validated questionnaire which includes psychological, physical, and sexual violence information^13^. The data collected includes information on sociodemographic (municipality and community of residence, age, years of schooling, and income); behavior (smoking, alcohol and drugs, contraception, age at first sexual intercourse, and number of sexual partners in life and in the last 12 months); clinical health (sexually transmitted infections (STI) and history of miscarriage); partner’s behavior (multiples sexual partners, drug abuse, and previous imprisonment); previous experience of violence in the family (related to alcoholism, drug abuse, involving children, or with people outside the family); and knowledge about the Maria da Penha Law - Law 11,340, of August 7, 2006 (Brazilian law that creates mechanisms to restrain domestic and family violence against women)^14^.

The outcome variables were the prevalence of psychological and/or physical violence and sexual violence caused by the intimate partner. The sample size was calculated to estimate the prevalence rate of violence with a 95% confidence interval of bilateral size of 0.5%. A 9.8% rate of physical violence was used as the basis for calculation, as it was the lowest prevalence found^7^, accepting a variability of +/- 4.0%, which generated a number of 193 women. Considering the possibility of losses, 20% was added to the total sample, which generated a number of 231 women.

Descriptive analysis was performed, including frequency distribution for qualitative variables, as well as calculation of mean and standard deviation (SD) for quantitative variables. To verify the association between sociodemographic, behavior, and clinical health, the chi-square test with Yates correction or Fisher’s exact test were performed when appropriate. Bivariate analysis was performed, using Pearson’s chi-square test, between the independent variables and the presence of violence to determine the value of statistical significance and selection of variables included in the logistic regression model. Variables with p-value ≤ 0.15 were included in the multivariate logistic regression model. Variables were considered significant when p-value was < 0.05.

This project was approved by the Research Ethics Committee of the Health Sciences Center of the Federal University of Espírito Santo, under Opinion no. 1252709/2015. All women were included in the study after signing the Informed Consent Form.

## RESULTS

A total of 219 women (94.8%) agreed to participate in the study. The mean age was 41.4 years (SD = 14.3 years), 45.2% was in the age group from 25 to 44 years old. A total of 83.8% lived in the rural area, 71.2% had less than eight years of schooling, 64.4% had a monthly income equal to or less than one Brazilian minimum wage, and 61.5% reported difficulty in accessing the health care services was mentioned. The prevalence rates of IPV were 59% (95%CI: 52.5–65.5) for psychological violence; 41% (95%CI: 34.5–47.5) for physical violence; and 8.2% (95%CI: 4.6–11.8) for sexual violence. These women also reported sexual violence perpetrated by other people; the rate was 14.1% (95%CI: 9.5–18.7).

In bivariate analysis, psychological violence was more frequent among women who had three or more sexual partners in life (p = 0.006) and in the last 12 months (p = 0.008). Physical violence was more frequent among those who had their first sexual intercourse at ≤ 15 years of age (p = 0.008), reported drug abuse (p = 0.014), and reported ≥ 3 partners in the last 12 months (0.008) (Table 1).

**Table 1:** Sociodemographic and behavioral variables associated with psychological and physical violence by intimate partner, in Quilombola women, in the State of Espírito Santo, Brazil, 2018. (n = 219).

Variable	n	%	Psychological violence					Physical violence				
	
			No		Yes		p	No		Yes		p
			
			n	%	n	%		n	%	n	%	
Age (years)							0.158					0.234
≤ 35	76	34.7	26	34.2	50	65.8		41	54.0	35	46.0	
> 35	143	65.3	63	44.0	80	56.0		89	62.2	54	37.8	
Education (years)							0.429					0.904
0–8	156	71.2	66	42.3	90	57.7		93	59.6	63	40.4	
≥ 9	63	28.8	23	36.5	40	63.5		37	58.7	26	41.3	
Monthly income^a^							0.344					0.377
≤ 1 Minimal wage	141	64.4	57	40.4	84	59.6		87	61.7	54	38.3	
> 1 Minimal wage	46	21.0	15	32.6	31	67.4		25	54.3	21	45.7	
Tobacco use							0.064					0.590
No	199	90.1	77	38.7	122	61.3		117	58.8	82	42.2	
Yes	20	9.1	12	60.0	8	40.0		13	65.0	7	35.0	
Alcohol abuse							0.813					0.459
No	168	76.6	69	41.0	99	59.0		102	60.7	66	39.3	
Yes	51	23.3	20	39.2	31	60.8		28	54.9	23	45.1	
Drug abuse							0.509					0.014
No	212	96.8	87	41.0	125	59.0		129	60.8	83	39.2	
Yes	7	3.2	2	28.6	5	71.4		1	14.3	6	65.7	
First sexual intercourse							0.219					0.008
≤ 15	80	36.5	28	35.0	52	65.0		38	47.5	42	52.5	
> 15	138	63.0	60	43.5	78	56.5		91	65.9	47	34.1	
#Sexual partners in life							0.006					0.262
≤ 2	106	48.4	53	50.0	53	50.0		67	63.2	39	36.8	
> 3	113	51.6	36	31.9	77	68.1		63	55.7	50	44.3	
#Sexual partners last 12 months							0.008					0.001
≤ 2	177	80.8	80	45.2	97	54.8		114	64.4	63	35.6	
> 3	40	18.2	9	22.5	31	77.5		14	35.0	26	65.0	

^a^ Brazilian minimum income (BMI) in 2018 = US $251

Table 2 shows partners’ behaviors and previous history of violence. Psychological violence was associated to previous interparental violence related to drug abuse (p = 0.014), family violence involving children (p = 0.026), and people outside the family (p < 0.001). Physical violence was associated to drug abuse (p = 0.005), previous imprisonment (p = 0.034), alcohol abuse (p < 0.001), drug abuse in their family (p = 0.005), and violence involving children (p < 0.001) and people outside the family (p = 0.003).

**Table 2:** Partner’s behaviors and history of violence in family associated with psychological and physical violence by intimate partner, in Quilombola women, in the State of Espírito Santo, Brazil. 2018. (n = 219)

Variable	n	%	Psychological violence					Physical violence				
	
			No		Yes		p	No		Yes		p
			
			n	%	n	%		n	%	n	%	
Partner has other partners							0.139					0.054
No	178	81.0	69	38.8	109	61.2		99	55.5	79	44.5	
Yes	13	6.0	4	30.8	9	69.2		9	69.2	4	30.8	
No partner	28	13.0	16	57.1	12	42.9		22	78.6	6	21.4	
Partner using drugs							0.141					0.005
No	181	82.6	70	38.7	111	61.3		106	58.7	75	41.3	
Yes	10	4.4	3	30.0	7	70.0		2	20.0	8	80.0	
No partner	28	13.0	16	57.1	12	42.9		22	78.6	6	21.4	
Partner in prison							0.158					0.034
No	174	79.4	67	38.5	107	61.5		101	58.0	73	42.0	
Yes	17	7.6	6	35.3	11	64.7		7	41.2	10	58.8	
No partner	28	13.0	16	57.1	12	52.9		22	78.6	6	21.4	
Violence in family related to alcoholism							0.069					0.000
No	155	70.8	69	44.5	86	55.6		106	68.4	49	31.6	
Yes	64	29.2	20	31.2	44	69.8		24	37.5	40	63.5	
Violence in family related to drug abuse							0.014					0.005
No	199	90.9	86	42.3	113	57.7		124	61.3	75	38.7	
Yes	20	9.1	3	15.0	17	85.0		6	30.0	14	70.0	
Violence in family involving children							0.026					0.000
No	194	88.6	84	43.3	110	56.7		124	63.9	70	36.1	
Yes	25	11.4	5	20.0	20	80.0		6	24.0	19	76.0	
Violence in family involving other people							0.000					0.003
No	178	81.3	85	47.4	93	52.6		114	64.0	64	36.0	
Yes	41	18.7	4	9.7	37	90.3		16	39.0	25	61.0	
Having already heard about Maria da Penha Law							0.840					0.854
Yes	211	96.3	86	40.7	125	59.3		125	59.2	86	40.8	
No	8	3.7	3	37.5	5	62.5		5	62.5	3	37.5	

The final model of logistic regression for psychological violence shows that IPV remained associated with having three or more sexual partners in life, when compared to those who had up to two partners (OR = 2.2; 95%CI: 1,22–3,97, p = 0.009), and previous violence involving people outside of family increased the chance of IPV more than nine times (OR = 9.66; 95%CI: 3.21–29.14; p < 0.001). Regarding physical violence, the association with no use of barrier contraception (OR = 8.06; 95%CI: 1.20–52.63; p = 0.031), having partner with other sexual partners (OR = 8.33; 95%CI:1.33–55.55; p = 0.024), having 3 or more sexual partners in the last 12 months (OR = 3.25; 95%CI: 1.41–7.47; p = 0.006), partner using illicit drugs (OR = 22,00; 95%CI: 2.4–200.18; p = 0.006), and alcoholism in the family (OR = 3.68, 95%CI: 1.73–7.82; p = 0.001), increased the chance of IPV in the final logistic model.

Table 3 and 4 show demographics and behavioral characteristics associated with sexual violence in bivariate analysis. IPV was associated to drug abuse (p = 0.046), first sexual intercourse ≤ 15 years old (p < 0.001), miscarriage (p = 0.029); previous STI (p = 0.046), history of partners drug abuse (p = 0.037), and gynecological symptoms (p = 0.025).

**Table 3:** Sociodemographic and behavioral variables associated with sexual violence by intimate partner, in Quilombola women, in the State of Espírito Santo, Brazil, 2018. (n = 219).

Variable	Sexual Violence						

	Total		No		Yes		p
		
	n	%	n	%	n	%	
Age (years)							
≤ 30	58	26.5	56	96.6	2	3.4	0.123
> 30	161	73.5	145	90.1	16	9.9	
Education (years)							
0 a 8	156	71.2	144	92.3	12	7.7	0.655
≥ 9	63	28.8	57	90.5	6	9.5	
Monthly income^a^							
≤ 1 BMI	141	75.4	129	91.5	12	8.5	0.666
> 1 BMI	46	24.6	43	93.5	3	6.5	
Tobacco use							
No	199	90.9	182	91.5	17	8.5	0.582
Yes	20	9.1	19	95.0	1	5.0	
Alcohol abuse							
No	168	76.7	154	91.7	14	8.3	0.911
Yes	51	23.3	47	92.2	4	7.8	
Drug abuse							
No	212	96.8	196	92.5	16	7.5	0.046
Yes	7	3.2	5	71.4	2	28.6	
Age of first sexual intercourse							
<15	80	36.7	70	87.5	10	12.5	0.083
≥15	138	63.3	130	94.2	8	5.8	
Condom use							
Yes	49	22.50	44	89.8	5	10.2	0.564
No	159	72.90	146	91.8	13	8.2	
No partner	10	4.60	10	100			
# Sexual partners in life							
≤ 2	106	48.4	99	93.4	7	6.6	0.399
> 3	113	51.6	102	90.3	11	9.7	
# Sexual partners last 12 months							
≤ 2	177	81.6	162	91.5	15	8.5	0.840
> 3	40	18.4	37	92.5	3	7.5	

^a^ Brazilian minimum income (BMI) in 2018 = US $251.

**Table 4:** Partner’s behaviors and history of violence in family associated with sexual violence by intimate partner, in Quilombola women, in the State of Espírito Santo, Brazil, 2018. (n = 219)

Variables	Sexual Violence						p

	Total		No		Yes		
		
	n	%	n	%	n	%	
Miscarriage							
No	158	72.1	149	94.3	9	5.7	0.029
Yes	61	27.9	52	85.2	9	14.8	
STI							
No	212	96.8	196	92.5	16	7.5	0.046
Yes	7	3.2	5	71.4	2	28.6	
Partner has other partners							
No	178	81.3	164	92.1	14	7.9	0.620
Yes	13	5.9	11	84.6	2	15.4	
No partner	28	12.8	26	92.9	2	7.1	
Partner using drugs							
No	181	82.6	168	92.8	13	7.2	0.037
Yes	10	4.6	7	70.0	3	30.0	
No partner	28	12.8	26	92.9	2	7.1	
Partner in prison							
No	174	79.5	161	92.5	13	7.5	0.337
Yes	17	7.8	14	82.4	3	17.6	
No partner	28	12.8	26	92.9	2	7.1	
Partner with STI							
No	187	85.4	171	91.4	16	8.6	0.807
Yes	4	1.8	4	100.0			
No partner	28	12.8	26	92.9	2	7.1	
Gynecological symptoms							
No	91	41.6	88	96.7	3	3.3	0.025
Yes	128	58.4	113	88.3	15	11.7	
Violence in family related to alcoholism							
No	155	70.8	143	92.3	12	7.7	0.689
Yes	64	29.2	58	90.6	6	9.4	
Violence in family related to drug abuse							
No	199	90.9	184	92.5	15	7.5	0.247
Yes	20	9.1	17	85.0	3	15.0	

The final model of logistic regression for sexual violence showed that IPV remained associated with miscarriage (OR = 3.6; 95%CI: 1.28–10.22; p = 0.016), partner using drugs (OR = 6.2; 95%CI: 1.33–28.99; p = 0.020), and gynecological symptoms (OR = 3.9; 95%CI: 1.03–14.84; p < 0.045) (Table 5).

**Table 5:** Logistic regression analysis of associated factors to sexual violence by an intimate partner, in Quilombola women, in the State of Espírito Santo, Brazil, 2018.

Independent variables	Adjusted OR	95%CI	p
Age ≤ 30 years	0.3	0.066–1.637	0.174
Illicit drug abuse	2.2	0.243–19.535	0.486
First sexual intercourse younger than 15 years	2.4	0.850–6.808	0.980
Previous miscarriage	3.6	1.276–10.224	0.016
Previous STI	1.9	0.182–19.987	0.591
Partner using drugs	6.2	1.326–28.990	0.020
Gynecological symptoms	3.9	1.034–14.837	0.045

## DISCUSSION

This is the first study that addresses intimate partner violence (IPV) in women living in Quilombola communities in Espírito Santo. The results showed that violence is a structural situation in these women lives since high prevalence rates of violence were reported. The women were frequently exposed to more than one kind of violence; these data agree previous studies carried out in Brazil and in other countries^15,16^.

The data on psychological violence found in our study was similar to the ones described in systematic reviews about IPV in Latina women^17^ and from low- and middle-income class that included Latin American women^15^. These data show the importance of understanding the factors that are barriers for women to seek support and emphasize the need to elaborate strategies to control IPV, since it is frequently associated with less decision-making power among victims^18^. This is the most neglected type of violence, since it is usually the first form to occur, it manifests slowly and quietly, and sometimes it is not recognized by the victim. It can progress in intensity and consequences, and it can lead to physical aggressions^19^.

Regarding the physical violence described in this study, the prevalence was 41%, higher that the ones described in other Brazilian studies conducted in women seeking care at health units^,8,9^. It is important to highlight that Quilombola women present a different profile from women attending public health clinics in Brazil since they live in rural areas and have less access to education and health services. Low education, socioeconomic status, and rural areas are risk factors associated with IPV^20^.

The Maria da Penha Law was published to prevent domestic and family violence against women in Brazil, adopting preventive measures and guaranteeing the security of victims^14^. This law aims to reduce the rates of domestic of violence and most of the participants in this study were aware of it (96.3%), but even so, we observed high prevalence of psychological and physical violence. A study that proposed to compare the mortality rate caused by IPV in the periods before (2001 to 2006) and after (2007 to 2011) the Maria da Penha Law was in force showed no decrease in female mortality^21^. Another study documented that women who previously reported physical violence were those who had a higher risk of death due to violence^22^. This situation worsens when Black women living in rural areas are involved, due to the difficulty of access to effective information and to public services. The lack of access affects their right to citizenship, either through gender violence within the *quilombo*, where women learn that men are in charge, or caused by their vulnerability due to low socioeconomic conditions^12^. However, it is important to point out that the Maria da Penha law is an achievement in the policies focused on violence against women.

Our study reports that having three or more sexual partners increased the chance of violence. This result agrees with a study carried out in South Africa, which associated an increased risk of violence with having more than one partner per year^23^. Study carried out in some areas of the United States also reported that the increased risk of violence is linked with the higher number of sexual partners^24^. The history of violence inside the family environment related to alcohol abuse was also described in this study as responsible for increased exposure of women to violence, suggesting the idea of transmitting violence to next generation. In Nigeria, women who were exposed to interparental violence were four times more likely to suffer IPV than women not exposed to it^25^. Another study showed an association between interparental violence within the perpetrator’s family and the increased likelihood that he would be in involved in IPV^16^. These reports highlight the cyclical nature of violence, which can be “inherited,” making these women more vulnerable.

A study carried out in Brazil reported, based on the speeches by the Quilombola women regarding their perception of IPV, that women learn and internalize modes of submission due to cultural transmission throughout their life. Women are taught that one must obey their male partners and bear all of it for the preservation of the family; data showed IPV associated with feelings of guilt and shame of making it public^26^. Considering that a striking feature of Quilombola communities in Brazil is the fact that most of them live in communities far from the urban area, the issue of violence becomes invisible to external eyes.

The abuse of alcohol and illicit drugs are also important proxies of IPV. Our study showed that partner using illicit drugs increased the women’s chance of suffering physical violence. This association was also found in other studies^27,28^. Women who are victims of violence indicated the use of alcohol and/or other drugs as a triggering factor for the aggressions, since they occurred when the partner was under the influence of these substances. A previous study carried out in Quilombola communities described the great influence of alcohol and illicit drugs as a trigger for violence, and that the use of these substances was high in these communities^26^. This study also identified a high frequency of women who did not use any contraceptive method and a low frequency of those who used condoms as contraception, which may be influenced by the partner decision of using it. A study carried out in New York described that women who were highly dependent on their partners and feared abuse related to condom negotiation had a higher frequency of unprotected sex^29^.

The information bias could be one of the limitations of this study. The frequency of IPV may have been underestimated due to the false answers due to shame and fear of prejudice. However, in-person interviews in a private room may have minimized this bias. Other limitation was the cross-sectional design since it is not the ideal model for assessing factors associated with the outcome. Nevertheless, the good quality of the collected data and the high rate of answers helped to reduce this limitation.

Our results highlight the situation of violence in Quilombola women. It is important to note that vulnerable conditions contribute to the outcome, since the lack of access to information, knowledge of their rights, and assistance from health, legal, and social services increase the exposure of these women to violence. This scenario contributes to understanding the violence against women in an articulated perspective of historic background, of gender and race inequalities, and of community and interpersonal dimensions^30^. Awareness must be raised on the importance of reducing social and racial inequities for interrupting the culture of violence against women. We must challenge the common acceptance that domestic violence is a private topic and can be resolved in a private setting and remember that women face violence and intimidation within their own families from their own partners. Above all, me must increase visibility to the discussion about violence against the most vulnerable social segments, such as the Quilombola women, and implement prevention and assistance strategies, considering different axes of violence from the perspective of human rights and intersectionality.
